# Mechanism of Self-Healing Hydrogels and Application in Tissue Engineering

**DOI:** 10.3390/polym14112184

**Published:** 2022-05-27

**Authors:** Liang Quan, Yuan Xin, Xixi Wu, Qiang Ao

**Affiliations:** NMPA Key Laboratory for Quality Research and Control of Tissue Regenerative Biomaterial & Institute of Regulatory Science for Medical Device & National Engineering Research Center for Biomaterials, Sichuan University, Chengdu 610064, China; quanliang@stu.scu.edu.cn (L.Q.); xinyuanscu@stu.scu.edu.cn (Y.X.); wuxixi9997@163.com (X.W.)

**Keywords:** hydrogels, self-healing, mechanism, tissue engineering application

## Abstract

Self-healing hydrogels and traditional hydrogels both have three-dimensional polymeric networks that are capable of absorbing and retaining a large amount of water. Self-healing hydrogels can heal and restore damage automatically, and they can avoid premature failure of hydrogels caused by mechanical damage after implantation. The formation mechanism of self-healing hydrogels and the factors that hydrogels can load are various. Researchers can design hydrogels to meet the needs of different tissues through the diversity of hydrogels Therefore, it is necessary to summarize different self-healing mechanisms and different factors to achieve different functions. Here, we briefly reviewed the hydrogels designed by researchers in recent years according to the self-healing mechanism of water coagulation. Then, the factors for different functions of self-healing hydrogels in different tissues were statistically analyzed. We hope our work can provide effective support for researchers in the design process of self-healing hydrogel.

## 1. Introduction

Hydrogels with adjustable physical and chemical properties are composed of 3D polymeric networks [1]. Hydrogels are similar to the structure and function of the extracellular matrix, which can absorb and retain a large amount of water without dissolving in a swelling state [2]. Therefore, hydrogels have long been widely used in tissue repair, drug delivery, and other fields [3,4,5]. Due to the complex dynamic physiological and mechanical environment inside the human body, the hydrogel will appear with minor defects after implantation. Due to the irreversibility of the conventional hydrogel after destruction, these minor defects will gradually increase and merge into large cracks, resulting in material failure [6,7,8]. Consequently, to improve the practical life of implanted hydrogel, it is necessary to design a self-healing hydrogel.

Self-healing material is defined as the material that can heals and restores damage automatically. Self-healing hydrogels can achieve self-healing through external stimuli (light, heat, pH) or the interaction of mutual covalent and noncovalent bonds of functional groups in hydrogels after damage [9,10,11]. The self-healing properties of self-healing hydrogels improve the fatal shortcomings of hydrogels that cannot recover spontaneously after damage, greatly promote the development of hydrogels to multifunctional composite hydrogels, and further broaden the application of hydrogels in the field of biomedicine.

The related research on self-healing hydrogels is increasing. The self-healing mechanisms of self-healing hydrogels of different researchers and the application directions of hydrogels are also different. In this review, we review the research results of self-healing hydrogels in recent years according to the self-healing mechanism of hydrogels. Then the different applications of self-healing hydrogels in tissue engineering were summarized, and the functional factors of self-healing hydrogels in various applications were generalized. We hope that the summary of this paper can provide a theoretical reference for researchers to develop more novelty-specialized intelligent hydrogels.

## 2. Mechanism of Self-Healing Hydrogels

The self-healing mechanisms of hydrogels are mainly based on the reversibility of their cross-linking structures, which often involve dynamic covalent bonds [1,2] (such as Schiff base bond, borate ester bond [3], Diels–Alder reaction [4], and disulfide bond [5] and dynamic noncovalent bond interactions (such as hydrogen bond interaction [6], ion interaction (metal coordination) [7,8], host–guest interaction [9,10], hydrophobic interaction [11]). Hydrogels’ stability, self-healing ability, and mechanical properties are directly related to the number and strength (or type) of chemical bonds used in the synthesis of hydrogels. Therefore, researchers need to understand the self-healing mechanism of different hydrogels to design hydrogels with varying self-healing effects.

The covalently cross-linked hydrogel polymer network synthesized by the traditional chemical method is irreversible and easy to be fatigued or damaged in use. Because the dynamic covalent bond is reversible as a process of ‘fracture-formation’ in a mild environment, it is often used to construct self-healing hydrogels. Dynamic noncovalent bond interaction is a kind of physical interaction with natural reversibility and stability of dynamic fracture recombination. It is a good choice for designing self-healing hydrogel.

### 2.1. Schiff Base

**Figure 1:**
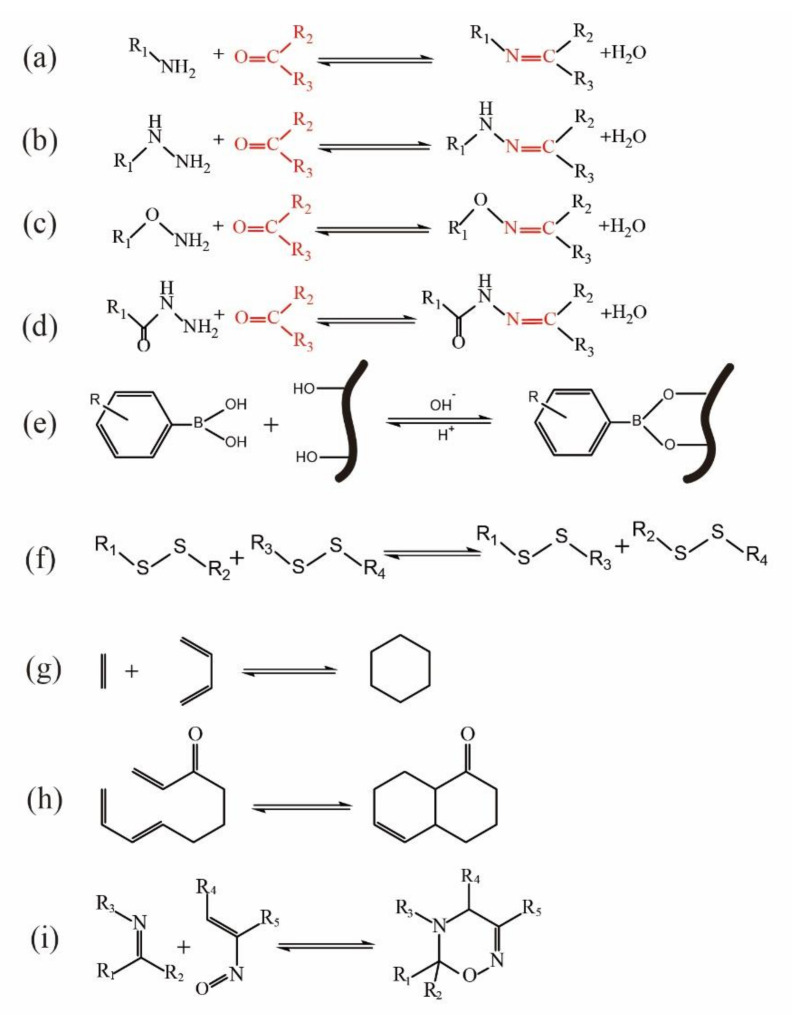
Self-healing hydrogels based on dynamic covalent bonds: (**a**) The imine formed between aldehyde and imine; (**b**) The hydrazone formed between aldehyde and hydrazide; (**c**) The oxime formed between aldehyde and aminooxy; (**d**) The acylhydrazone formed between aldehyde and acylhydrazide (The red color represents the substance with active carbonyl functional groups); imine or methylimine characteristic groups (-RC=N-). (**e**) The complexation equilibrium of borate ester bond; (**f**) The disulfide bonds addition reaction; (**g**) The normal electron-demand DA reaction; (**h**) The intramolecular DA reaction; (**i**) The hetero-DA reaction.

German chemist Hugo Schiff discovered the imine bond in 1864, so amine-based compounds are usually called “Schiff bases” [12]. Schiff base is considered a strong covalent bond (150 kcal mol^−1^) [13], including imine (Figure 1a), oximes (Figure 1b), hydrazones (Figure 1c) and hydrazine (Figure 1d). In Schiff base reaction, the amine nitrogen (a nucleophile) attacks the electrophile carbonyl atom in the aldehyde to yield a double nitrogen–carbon bond (Schiff base). In addition, the Schiff base can still go back to amines and the functional carbonyl groups through hydrolysis. Consequently, the Schiff base reaction may form a dynamic equilibrium under physiological conditions. In recent years, Schiff bases, as a class of chemical bonds capable of dynamic reversible fracture recombination, have been widely used in hydrogel networks to construct self-healing hydrogels [14]. The acylhydrazone bond is usually synthesized by the condensation reaction of hydrazine and aldehyde [15,16]. The acylhydrazone bond is exceptionally similar to the imide bond, but the acylhydrazone bond can spontaneously form under physiological conditions (the rate is much lower than that in an acidic environment) [16,17]. Polymers possess amino-rich such as chitosan and polyethyleneimine, combine with polymers containing aldehyde and ketone groups, and obtain unique self-healing properties by the dynamic reversibility of imide and acylhydrazone bonds.

ε-poly(L-lysine) carbon dot (PL-CD) with a large number of amino groups on the surface and oxidized dextran (ODA) were cross-linked by Schiff base to form hydrogels [18]. In addition to excellent self-healing properties, PL-CD also had excellent antibacterial properties. As the contacting and releasing antibacterial action of the PL-CD@ODA hydrogel, 10% CFU/mL of 10^7^ *S. aureus* was killed after 10 min of contact. In addition, PL-CD@ODA hydrogels showed flexible injection and strong self-healing properties after severe damage. Complete healing can be achieved in a few seconds when 1000% shear stress is applied to the hydrogel. Baolin Guo et al. [19] successfully synthesized injectable self-healing conductive hydrogel composed of dextran grafted aniline tetramer-grafted-4-formylbenzoic acid (Dex-AT-FA) and *N*-carboxyethyl chitosan (CECS). Grafted aniline oligomers provide sufficient electrical activity and conductivity for hydrogels (10^−2^ ms/cm); dynamic cross-linked network formed by Schiff base bond provides excellent self-healing ability for hydrogels. The hydrogel with enough in vivo injection and degradation can encapsulate different types of cells, and the released cells still show continuous proliferation ability.

### 2.2. Borate Ester Bond

Boronic acid can accept electron pairs; it can form complexes with hydroxide or electron-donating groups containing oxygen or nitrogen atoms. In an aqueous solution, boronic acid co-exists in its neutral state or is bound to a hydroxide ion, and its sp^2^ and sp^3^ hybrid orbitals adopt trigonal planar and tetragonal geometries. Therefore, Boronic acid forms a borate ester bond with 1,2-diols or 1,3-diols in an aqueous solution or in its bulk form or an organic solvent [20]. The dynamic equilibrium of borate ester bond reversibly formed or broken is affected by pH, heat, aqueous media and some biomolecules. For this reason, boronic acid can be applied to sensors that can selectively detect the biomolecules including saccharides, such as glucose [21,22], or can be used as a component in the drug delivery systems [23,24], and self-healing materials.

Phenylboronic acid and cis-diol-modified polyethylene glycol form boric acid ester bonds to cross-link into pH-responsive hydrogels [25]. These gels also exhibit size-dependent controlled release of proteins encapsulated within the network and the glucose-responsive release of larger proteins.

Dynamic borate hydrogel can also improve the bioavailability of poorly water-soluble drugs (such as curcumin) [26]. Ruihao Pan et al. developed a multi-responsive self-healing hydrogel for the controlled release of curcumin by using a dynamic borate ester bond. Hydrogels were synthesized by coupling curcumin with polyvinyl alcohol using a polymer containing phenylboronic acid. The synthesized curcumin-loaded hydrogels were self-healing due to the dynamic nature of borate bonds; the variety of stimuli can change the equilibrium transfer between borate and boric acid, thereby affecting the release rate of curcumin to achieve drug-controlled release.

### 2.3. Disulfide Bond

The disulfide bonds (R–S–S–R) plays a main role in the folding of cysteine residues to protein [27,28]. Therefore, disulfide bonds are widely distributed in nature. Thiol-disulfide exchange reactions can occur between thiolates and disulfides. In addition, thiols can be oxidized to disulfides, and disulfides can be reduced to thiols. Thus, the reaction that forms disulfide bonds under physio-logical conditions is dynamic and reversible [29]. The homolytic dissociation energies of disulfides generally are low (50–65 kcal mol^−1^). The various nature of the groups flanking the disulfide bonds make the bonds respond to different stimuli (such as light, heat, mechanical force, and changes in the pH or redox state) [30]. Consequently, disulfides can tune the properties of materials under mild reaction or stimulus conditions. The ease with which thiols can be converted to disulfides, and vice versa, renders disulfide bonds containing polymers as dynamic self-healing. Hydrogels cross-linked by disulfide bonds exhibit self-healing properties in addition to rapid gel formation [31].

Xin Zhang et al. [32] constructed injectable and rapid self-healing protein hydrogel by recombining the disulfide bond in Bovine serum albumin (BSA). By reducing the disulfide bond in BSA protein, the structure of BSA protein was unfolded. Without changing the secondary structure of BSA, the disulfide bond between each protein molecule was recombined to form a nontoxic, injectable, and rapid self-healing hydrogel. H_2_O_2_ can accelerate thiol oxidation at the damaged interface of BSA protein hydrogel so that the hydrogel can be wholly repaired within 1–2 min, and the repair efficiency reaches 100%. BSA protein hydrogel showed good fluid performance at 39% strain, which a pinhole injector could extrude. In another study, Hansen Yu et al. [33] prepared injectable thermal responsive hydrogels that self-healing under weak acidic to alkaline conditions. The dynamic hydrogels prepared by mixing thiol functionalized F127 with disulfide-modified Polyethylene glycol(PEG) still have thermal responsiveness and undergo sol-gel transition under temperature change. In addition, hydrogels achieve self-healing under neutral or even mildly acidic conditions because the cyclic tension of cyclic disulfides increases the reactivity of disulfide bonds.

### 2.4. Diels-Alder(DA)

Otto Diels and his student, Kurt Alder, described the Diels–Alder (DA) reaction in 1928 [34]. DA reaction is stereoselective, atom economical, and highly efficient; it is one of the most powerful methods to synthesize unsaturated six-membered rings [35]. Six π electrons rearrange to form a six-membered ring product by DA reaction (Figure 1g–i). DA reaction readily proceeds in water; it does not require expensive and potentially toxic solvents; and it can react at room temperature. Additionally, The DA reaction greatly accelerated in water by hydrogen bonding to the activated complex and enforced hydrophobic interactions between the reactants [36,37,38,39]. DA reaction includes normal electron-demand DA reaction, intramolecular DA reaction and hetero-DA reaction. Due to thermal responsiveness, covalent bond strength, high selectivity, high chemical yield, and lack of byproducts, DA reaction is widely used to prepare dynamic covalent hydrogels [40]. In addition, DA reaction belongs to click chemistry, which is considered a kind of biocompatibility reaction and can realize substrate binding to specific biomolecules.

Zhao Wei et al. [41] prepared dextran-l-poly (ethylene glycol) self-healing hydrogel by reversible DA reaction. The hydrogel was prepared using biocompatibility fulvene-modified hydrophilic dextran (Dex-FE) as the main polymer chain and dichloroacetic acid-modified polyethylene glycol (PEG-DiCMA) as the cross-linking agent to form the hydrogel network dextran-l-poly (ethylene glycol) self-healing hydrogel.

In addition to good biocompatibility, the dextran-l-poly (ethylene glycol) self-healing hydrogel was divided into two parts after 7 h to achieve self-healing at 37 °C. De-qiang Li et al. [42] also prepared self-healing pectin/chitosan hydrogel by Diels–Alder reaction. Compared with pectin/chitosan composite hydrogel prepared by pure physical interaction (electrostatic interaction and molecular entanglement), the cross-linking density of self-healing pectin/chitosan hydrogel was greatly improved by DA reaction, and it had pH responsiveness and good self-healing performance (bearing 500 g weight without damage). At the same time, self-healing pectin/chitosan hydrogel can achieve higher drug loading efficiency, and the drug release mechanism can be controlled by adjusting the cross-linking density.

### 2.5. Hydrogen Bond

**Figure 2:**
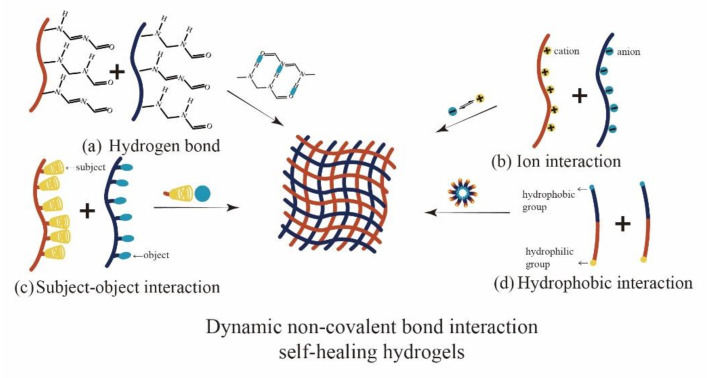
Self-healing hydrogels based on dynamic non-covalent bond interactions: (**a**) Hydrogen bond interaction; (**b**) Ion interaction (metal coordination); (**c**) subject-object interaction; (**d**) hydrophobic interaction.

Hydrogen bonds play a crucial role in the water solubility of substances [43], the stability of DNA double helix structure [44], the secondary and tertiary structures of proteins, and the properties of solid polymers [45]. Hydrogen bond (Figure 2a) is formed by the attraction between the hydrogen atom on the polar X–H bond and the lone pair electrons of another atom with strong electronegativity and small atomic radius (such as F, N, O). The hydrogen bonds between molecules are easy to form and have different properties at different temperatures and pH values, and the change is reversible. When the outside world destroys the hydrogel, the molecular chain of the polymer has fluidity under the action of hydrogen bonds, thus giving it self-healing property. The binding strength of different hydrogen bonds is significantly different but is weak (0.25–15 kcal mol^−1^) [46]. However, when there are many hydrogen bonds in hydrogels, hydrogen bonds have a significant improvement on the mechanical properties of hydrogels, and the formation and dissociation of hydrogen bonds are very fast, which can realize the rapid healing of hydrogels.

Chen et al. [47] synthesized tannic acid-lipoic acid (TATA) hydrogels by free radical nucleophilic addition reaction of the polyphenol-sulfur group and ring-opening polymerization of lipoic acid. Multiple hydrogen bonds formed between disulfide bonds, polyphenol residues, and carboxyl groups give hydrogel self-healing and injectability. Hydrogen bonding improves the self-healing time of TATA hydrogel and makes TATA hydrogel adhere to the wound quickly. X.X. Sun et al. [48] made self-healing PVA-agar hydrogel with excellent mechanical properties by hydrogen bond interaction. After freeze–thaw cycles, hydrogen bonds formed between polyvinyl alcohol (PVA) molecules and between PVA molecules and agar molecules so that the hydrogel has self-healing properties. The healing hydrogel can withstand 2.3 kg weight and 5.0 MPa tensile strength, and the elongation at break can reach 450%.

### 2.6. Ion Interactions

The reversible electrostatic interactions between oppositely charged moieties ionic bonds can help hydrogel realize the self-healing (Figure 2b) [49,50]. This interaction can occur between the transition metal ions with empty orbits and the groups containing lone pair electrons and between polymers with opposite charges and between the same charged polymers with opposite charges.

The ions interaction frequently makes the mechanical properties of hydrogels more prominent. Hamed Daemi et al. [51] synthesized alginate-based supramolecular polyurethane (ASPU) with adjustable mechanical properties by the ion interaction between polyanionic alginate and polycationic polyurethane. The mechanical properties of ASPU can be adjusted by alginate content in an extensive range, and the tensile strength and Young’s modulus can reach 48 MPa and 93 MPa, respectively. In addition, ASPU has good self-healing ability and is divided into two parts of hydrogels, which can be reconnected after the 30 s of contact. W. Chen et al. [52] combined sodium carboxymethyl cellulose (CMC) with PAA-Fe^3+^ hydrogels to provide PAA-Fe^3+^ hydrogels with high tensile strength (4.42 MPa), high toughness (dissipation energy up to 1.98 MJ/m^3^), and good self-healing properties. The self-healing property of hydrogels is derived from the dynamic reconstruction of the metal ion coordination bond between polyacrylic acid (PAA) and CMC.

### 2.7. Host-Guest Interaction

As shown in Figure 2c, subject–object interaction generally occurs between two or more chemicals. These chemicals cause molecular assembly by binding to noncovalent complimentary inclusions, allowing one part (guest) to be physically inserted and contained in another part (host). Because this interaction is maintained through noncovalent interactions (such as hydrogen bonds and ionic bonding, van der Waals and hydrophobic interactions), cross-linking is reversible and can provide self-healing properties for hydrogels.

Zhijun Ren et al. [53] prepared double-net hydrogel by dynamic cross-linking of O-carboxymethyl chitosan (O-CMCS) and PVA main chain. The hydrogel has a variety of dynamic cross-linking (Schiff base, host–guest interaction, borate ester bond, and hydrogen bond). The hydrogel has rapid self-healing properties (self-healing without external stimulation for 10 s), tissue adhesion, water absorption, and mechanical properties. Beilin Zhang et al. [54] prepared QCS-CD-AD/GO hydrogel through host-guest interaction with quaternary ammonium chitosan grafted cyclodextrin (QCS-CD), quaternary ammonium chitosan grafted adamantane (QCS-AD), and graphene oxide grafted cyclodextrin (GO-CD) polymer solutions. The hydrogel had an excellent antibacterial activity of QCS and photo-thermal and electrical properties of reduced graphene oxide (rGO). The host–guest interaction between CD and AD provides self-healing properties for supramolecular hydrogels. In addition, the hydrogel has stable mechanical properties, good biocompatibility, and NIR radiation-induced antibacterial activity.

### 2.8. Hydrophobic Interactions

Hydrophobic interactions are as important as hydrogen bonds in protein folding, the properties of solid polymers, and the interactions between molecules in different solvents. Hydrophobic interactions refers to the phenomenon that hydrophobic groups close to each other to avoid water. Hydrophobic interactions are slightly stronger than hydrogen bonds, but the Hydrophobic interaction can be fine-tuned by changing the shape of hydrophobic area and the number of hydrophobic groups. Hydrophobic interactions occur in the aggregation of a hydrophobic surface or hydrophobic medium. Waterborne polymer chains aggregate and associate in an aqueous solution to form dynamic cross-linking points [55] (Figure 2d). The compact hydrophobic structure is easy to assemble, and the hydrophobic association area is rapidly reformed when destroyed. The reversible decomposition of hydrophobic interactions can provide self-healing ability for hydrogels.

Yuting Wang et al. [56] complexed sulfonated polyurethane (SPU) with PAA by Zn^2+^ to prepare SPU-PAA/Zn self-healing hydrogel films with high mechanical strength and excellent elasticity. The tensile strength and toughness of the hydrogel films are 7.1 MPa and 30.4 MJm^−3^, respectively. At the same time, the SPU-PAA/Zn hydrogel membrane can be wholly restored to the original shape after tensile stress release with a strain of less than 500%. The integrity and mechanical properties of the fractured hydrogel film can be self-repaired.

Siheng Li et al. [57] prepared PAAm/PAA-Fe^3+^/NaCl hydrogel by complexing polyacrylamide (PAAm) with Fe^3+^ chelating polyacrylic acid (PAA-Fe^3+^). The loading of NaCl in the hydrogel can generate hydrophobic regions to improve its mechanical strength and elasticity and endow the hydrogel with ionic conductivity up to 100% (0.72 S/m^−1^). Due to the hydrogen bond and coordination interaction, as well as the synergistic effect of hydrophobic regions, the synthesized PAAm/PAA-Fe^3+^/NaCl water.

## 3. Application of Self-Healing Hydrogels in Tissue Engineering

Self-healing hydrogel has good self-healing, fatigue resistance, and reusability and has hydrophilicity and responsiveness to environmental stimuli of conventional hydrogels. With researchers’ in-depth study of self-healing hydrogels, the application fields of self-healing hydrogels have significantly been expanded, showing excellent development potential in wound dressings and drug delivery, tissue engineering, bionic electronic skin wearable electronic equipment, and other fields. In this section, we summarize the recent application of self-healing hydrogels in tissue engineering, mainly including the self-healing mechanism of hydrogels, some mechanical properties, induction factors, and the characteristics of hydrogels. The recent research progress of hydrogels in bone, cartilage, skin, cardiovascular, sensor and other significant fields was summarized in the following contents.

### 3.1. Bone Repair

Through intervention, bone defects greater than the critical defect value caused by diseases such as bone tumors and fractures can be repaired [58]. As a new biomaterial with controllable mechanical properties and biocompatibility, hydrogel is widely used in bone tissue engineering (BTE) as a scaffold for growth factor transport and cell adhesion (Table 1). In bone tissue engineering, idealized hydrogel scaffold materials could provide three-dimensional living space for cells and regulate the morphology and function of tissue engineering cells. The hydrogel can effectively support protein absorption and cell adhesion so that cells grow following the prefabricated three-dimensional scaffold and are replaced by bone cells during the gradual degradation of the hydrogel to achieve bone repair [59].

M. J et al. [60] crosslinks *N*-hydroxysuccinimide-poly(2-oxazoline)s (Pox-NHS) and amine-poly(2-oxazoline)s/alendronate-poly(2-oxazoline)s (Pox-Ale) through Ca^2+^ interact to form a hydrogel network. In addition, Ca^2+^ can promote the repair of bone tissue to some extent. The alendronate-functionalized polymer has strongly reinforced the adhesive properties of the hydrogels. Pox-Ale@POx-NHS hydrogel has great potential in bone tissue bonding. Additionally, W. Huang et al. [61] increased catechol group on chitosan by chemical grafting. The catechol-conjugated chitosan (CS–C) and dialdehyde cellulose nanocrystal (DACNC)-formed hydrogel was by mild Schiff base reaction, catechol enabled the hydrogel to have adhesion, hemostasis, and defect repair functions.

**Figure 3:**
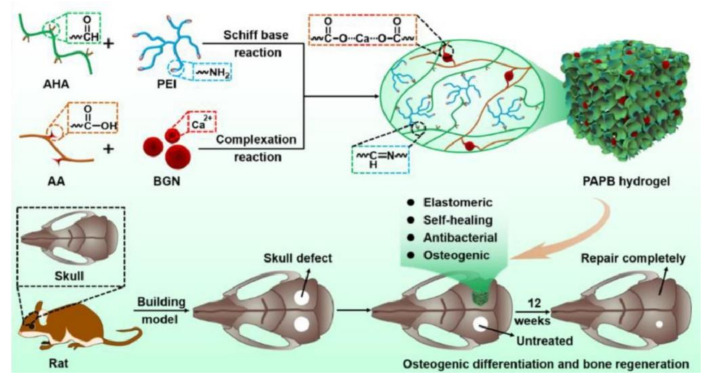
Elastomeric self-healing antibacterial bioactive nanocomposites scaffolds for treating skull defect [63]. Reproduced with permission from Li, Y, Appl. Mater. Today; published by Elsevier, 2022.

Researchers also added influence factors to the three-dimensional structural system of hydrogels to promote bone tissue repair. L. Ma et al. [62] doped hydroxyapatite in the oxidized alginate@carboxymethyl chitosan hydrogel as an induction factor for bone repair to improve bone repair performance (Figure 3). Different from the physical doping of the former, Y. Li et al. [63] used aldehyde Hyaluronic acid (AHA)@polyethyleneimine (PEI)@Acrylic acid (AA) as the carrier to add nano bioactive glass (BGN) by Schiff base cross-linking. BGN not only added the characteristics of bone differentiation and bone regeneration to hydrogels but also enhanced the physical properties of hydrogels by the new network of BGN in hydrogels.

X. Lu et al. [64] prepared self-healing hydrogels by combining chitosan with silk fibroin (SF) through hydrogen bond, ion interaction (Mg^2+^), amide bond. Chitosan@SF hydrogels delivered and released recombinant human bone morphogenic protein-2 (rhBMP-2)/rat bone marrow-derived MSCs (rBMSCs) using the three-site network structure of CS @SF hydrogels. D. Li et al. [65] also achieved similar results on the matrix of Alginate dialdehyde@gelatin.

**Table 1:** Self-healing hydrogel for bone repair.

Hydrogel Substrate	Self-Healing Mechanism	Self-Healing Cycle	Inductor	Binding Mode	Mechanical Property	Characteristics of Hydrogels	Ref.
AHA@PEI/AA	Schiff base/ Ion interaction	Completely healed after 12 h	Nanoscale bioactive glass	Schiff base	2000 Pa (at 88% Compression stress strain)	Antibacterial/enhanced osteogenic differentiation, skull regeneration/Drug delivery	[63]
CS/Silk fibroin(SF)	Hydrogen bond/ Ion interaction/ Amide bond	/	rhBMP-2/rBMSCs/Mg^2+^	Immobilized with structure	300 MPa (Young’s modulus)	Immobilization cells delivery and implantation of stem cells/Drug delivery	[64]
Oxidized pullulan/poly(ethylene glycol)-Dex	Hydrazone	Two-part distribution uniform within 24 h	Dexamethasone	Hydrazine	2.53 kPa (average storage modulus)	Antioxidant/anti-inflammatory/cell proliferative	[66]
POx-NHS@amine-poly(2-oxazoline)s/ POx-Ale/Ca^2+^	Hydrogen bond/ Ion interaction	recovery 107% within 10 min	CaP/soluble Ca^2+^	Ion interaction	200 kPa (low storage moduli)	Bone cohesive	[60]
CS-C@DACNC	Schiff base	within 2 min	Catechol	Chemical grafting	1402.1 Pa (storage modulus)	Cohesive ability/promote bone repair	[61]
Alginate dialdehyde@gelatin	Ion interaction/ Schiff base	within 5 min	Demineralized bone matrix/BMSCs	Immobilized with structure	112 kPa (compressive strength)	Injectable/osteocalcin and VEGF highly expressed/Bone regeneration effect and bone defect repair	[65]
Oxidized alginate@carboxymethyl chitosan	Schiff base	Two-part distribution uniform within 1 h	Hydroxyapatite	Schiff base	800 Pa (storing elastic deformation energy)	Potential bone regeneration effect/Bone defect repair	[62]

### 3.2. Cartilage Repair

Osteoarthritis and sports injury can cause articular cartilage lesions, but cartilage’s limited internal healing ability will lose cartilage [67,68]. The biomimetic microenvironment of self-healing hydrogel can promote cell migration, proliferation, and angiogenesis to repair cartilage tissue (Table 2). Articular cartilage needs more frequent mechanical stress than bones, so hydrogel used in cartilage repair needs better mechanical new energy and self-healing performance. It can also match the dynamic loading microenvironment of natural cartilage, providing more possibilities for cartilage tissue repair.

To meet the specific needs of hydrogel in cartilage repair, S. Maiz-Fernández et al. [69] selected chitosan and hyaluronic acid (HA) to construct hydrogel network by electrostatic interaction so that it has the possibility of loading a variety of drugs (the study also loaded with diclofenac and rifampicin). P. Baei et al. [70] introduced catechol into sulfated alginate (SAlg) to bind chitosan (CS), which not only endows hydrogel cells with high affinity for adhesion, tissue integration, and bio-factor immobilization. Moreover, the hydrophobicity of cats reduced the swelling ratio of hydrogels and improved the mechanical properties.

**Figure 4:**
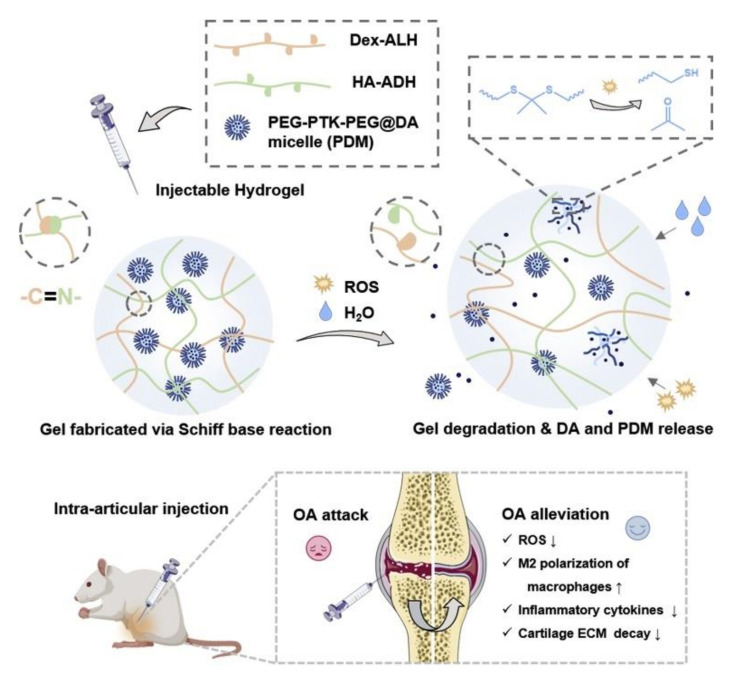
Schematic diagram showing the fabrication of hyaluronic (HA) and dextran (Dex) Schiff base hydrogel loaded with dexamethasone acetate (DA)-encapsulated PEG-PTK-PEG micelles (PDM) for osteoarthritis (OA) therapy in vivo. The hydrogel is swollen and/or degraded in OA joint, allowing the easier reaction between the PDM and ROS to release DA. They synergistically reduce the ROS concentration, upregulate the M2 polarization, downregulate the key inflammatory cytokines, and thereby achieve better therapy of OA in vivo [71]. Reproduced with permission from Zhou, T, Mater. Today Nano; published by Elsevier, 2022.

In addition to the selection and modification of the hydrogel network matrix, some researchers added new systems to the hydrogel network. T. Zhou et al. [71] used poly(ethylene glycol)-b-polythioketal-b-poly(ethylene glycol) (PEG-PTK-PEG) micelles as the carrier of dexamethasone (DA), combined with hydrazine grafted hyaluronic acid (HA-ADH) and aldehyde modified dextran (Dex-ALH) to achieve slow linear release of DA, reduce oxidative stress, and inhibit the occurrence of osteoarthritis (Figure 4). P. Wang et al. [72] added microcrystalline cellulose (MCC) to functional polyglutamic acid (γ-PGA) and sodium alginate (SA). MCC has many hydrogen bonds, which prolong the degradation time (125%) and reduce the expansion rate (470%).

**Table 2:** Self-healing hydrogel for cartilage repair.

Hydrogel Substrate	Self-Healing Mechanism	Self-Healing Cycle	Inductor	Binding Mode	Mechanical Property	Characteristics of Hydrogels	Ref.
HA@ Hydrazide	Hydrazone	2 h	Infliximab	Imine bonds	Tunable	Anti-inflammatory/ Drug delivery	[73]
HA-ADH@Dex-ALH	Schiff base	10 min	Dexamethasone	Loaded on micelles	0.39 kPa (storage modulus)	Alleviated osteoarthritis/ Preventing cartilage extracellular matrix degeneration	[71]
γ-PGA@SA	Schiff base/ Hydrogen bond	Short time	Microcrystalline cellulose	Hydrogen bond	60–144 kPa (mechanical strength)	Promote cartilage matrix deposition/ Enhancement of hydrogel system	[72]
CS@HA	Ion interaction	15 min	diclofenac and rifampicin	Electrostatic interaction	0.023 MPa (stress modulus)	Controlled release of drugs	[69]
SAlg@CS	Ion interaction	Fast (healing efficient 80%)	MSC and chondrocyte	Co-culture encapsulate	421.45 kPa (compressive strength)	Higher mechanical stability/ Cell adhesion/ Tissue integration	[70]

### 3.3. Skin Repair

Skin is the largest organ of the human body, which can regulate body temperature and feel external stimuli and prevent pathogens from invading the body [74]. However, direct contact with the outside world makes the skin vulnerable to damage. Self-repair function of the skin is often unable to repair itself when it exceeds the critical value [75]. Skin self-healing is often accompanied by scars and loss of appendages such as hair and sweat glands [76]. The inherent properties of hydrogel make hydrogel have potential application in wound dressings (Table 3). The hydrogel with self-healing property can achieve self-healing after material damage and maintain the protective effect of the material on the skin damage area.

**Figure 5:**
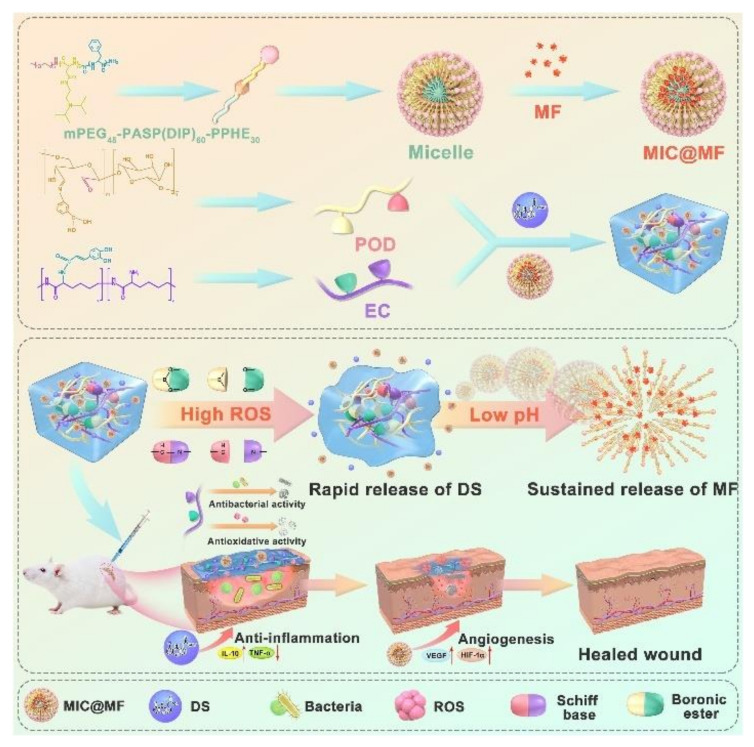
A spatiotemporal release platform based on pH/ROS stimuli-responsive hydrogel in wound repairing: The fabrication procedures of the DS&MIC@MF embedded POD/CE hydrogels, illustration of spatiotemporally drugs release behavior of the hydrogel, and the mechanism of the hydrogel for accelerating wound healing on the infected diabetic cutaneous wound model [77]. Reproduced with permission from Wu, Y, J. Control. Release; published by Elsevier, 2022.

Recently, Ye Wu et al. [77] designed caffeic acid-grafted ε-polylysine (CE) and phenylboronic acid-grafted oxidized dextran (POD) self-healing hydrogel with inherent antibacterial and antioxidant properties (Figure 5). Delytic sodium (DS) particles were directly embedded in the main chain of hydrogels, while MF was encapsulated in the core of micelles. Different binding modes make the release curves of mangiferin (MF) (sustained-release within 7 d) and DS (release 58.6% within 24 h) highly consistent with the healing process of the wound after infection. Zhixin Ling et al. [78] physically doped polydopamine nanoparticle (pDA-NPs) and enhanced the mechanical properties and expanded the pore size of CA hydrogel in the dynamic covalent cross-linked CA hydrogel based on Schiff base. The release of pDA-NPs effectively promoted the repair of large skin wounds, enhanced angiogenesis, and reduced scars.

In tissue engineering, conductive biomaterials can promote intercellular signal transduction and current transmission from external electrical stimulation, promoting cell migration and angiogenesis. HuanLei et al. [79] prepared this hydrogel by adding tannic acid (TA) and human-like collagen (HLC) into the dynamic cross-linking network of polyvinyl alcohol (PVA) and borax hydrogel, in which borax played the role of cross-linking agent and ionic conductor. The addition of HLC and TA changed the cross-linking density and pH value of hydrogels, thus adjusting the adaptability of the PVA-borax matrix and endowing it with hemostasis, antibacterial, anti-inflammatory, cell proliferation, and collagen deposition. Similarly, Zhulong Tu et al. [80] realized the conductive function of the material by adding graphene into the hydrogel system.

**Table 3:** Self-healing hydrogel for skin repair.

Hydrogel Substrate	Self-Healing Mechanism	Self-Healing Cycle	Inductor	Binding Mode	Mechanical Property	Characteristics of Hydrogels	Ref.
L-arginine-A@-CHO-PEG-CHO	Schiff-base	5 min	pDA-NPs	Physically doping	1.1 kPa (storage modulus)	Swelling capacity	[78]
POD-@DS&Micelles	Schiff base/ borate ester bond	2 min	MF/DS	Encapsulated into micelles	2 kPa (storage modulus)	pH/ROS dual-responsiveness/ Spatiotemporal delivery	[77]
PVA@borax hydrogel	Hydrogen bond/ Borate ester bond	Within 30 min	Tannic acid/HLC	Borate bonds	10 kPa (storage modulus)	Self-adaptive/Self-healing properties/Bioadhesion	[79]
Polypeptide@polydopamine	Schiff-base/ Hydrogen bond	60 min	Graphene oxide	Hydrophobic interaction/ Hydrogen bond	8 kPa (storage modulus)	Thermosensitive/Antibacterial/ Antioxidant/conductive	[80]
CMCS-CQD_AG_@ODex	Schiff base	3 h	carbon quantum dots (CQD_AG)_	Schiff base	11 kPa (storage modulus)	Antibiofilm/Low-drug resistance/Flexibility	[81]
PVA@silk fibroin@borax	Borate ester bond	30 s	Tannic acid	Hydrogen bond	4500 Pa (storage modulus)	Antibacterial/Flexibility/Plasticity/Bioadhesion/Easy stripping	[24]
COL-GG-PNIPAM-GO-borax	Diol-Borate ester bond	Within 3 min	/	/	5 kPa (storage modulus)	Conductive/Thermo and NIR sensitive/Accelerated healing	[82]
HA-PBA@TA/AgNP hydrogel	Borate ester bond	10 min	AgNP	Encapsulated into micelles	425 Pa (storage modulus)	Dual stimuli responsive/antibacterial/Anti-oxidative	[83]

### 3.4. Cardiac Repair

Due to insufficient blood supply, many cardiomyocytes will be lost, and relatively disordered fibrous tissues will occupy highly ordered cardiomyocytes. Excessive and sustained fibrosis deposition in the matrix disrupts electrical integrity and electrical signal conduction between health and infarction sites, leading to systolic and diastolic dysfunction and arrhythmia [84]. In these cases, reconstruction of expected electrical pulse propagation in fibrotic tissues has become the most critical issue in myocardial infarction repair [85]. Hydrogel-engineered heart patch can prevent heart failure after myocardial infarction and provide drugs or cells to promote damaged myocardial repair [86]. Cardiac pulsation can easily lead to hydrogel damage and premature loss (Table 4). Compared with traditional hydrogels, the drug delivery system’s self-healing hydrogels’ self-healing ability can ensure longer drug delivery and release time.

Rui Chen prepared a novel self-healing elastin to mimic peptide hydrogel (EMH) [87]. Polydopamine (PDA) nanoparticles loaded with salvianolic acid B (SaB) can deliver SaB locally and enhance the self-healing ability of hydrogel. Prehydrogel (SaB-PDA/pre-EMH) has excellent biocompatibility and low viscosity, making it suitable for myocardial injection. Once injected into the myocardial infarction (MI) region, SaB-PDA/pre-EMH can form SaB-PDA/pre-EMH with tremendous mechanical strength under the action of upregulated glutamine transferase (TGase) in cardiac tissue after myocardial infarction.

**Figure 6:**
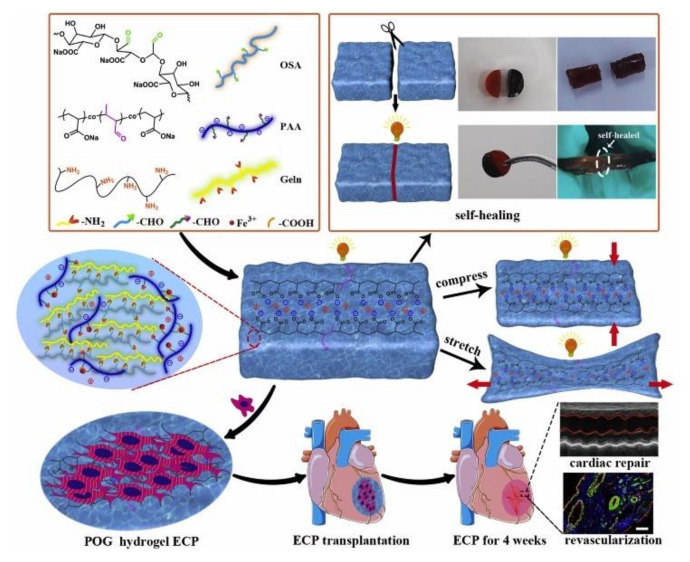
Schematic illustration about the fabrication of the tunable self-healing POG1 hydrogel and its application in myocardial infarction repair. Adapted with permission [88]. Reproduced with permission from Song, X, Biomaterials; published by Elsevier, 2021.

Conductive scaffold materials have a positive effect on the behavior of myocardial cells. Song, XP et al. [88] built PAA nanochannels inside porous ionic conductive hydrogel POG1 hydrogel, giving the hydrogel micro-heterogeneous electrical conductivity to make the cardiomyocytes (CM) inoculated in macroporous ionic conductive hydrogel (POG1) hydrogel show more oriented sarcomere (Figure 6). In addition, POG1 hydrogel has suitable scalability (>500% strain) and compression (>85% strain), and its modulus is similar to that of the mammalian heart (30–500 kPa, Young’s modulus).

### 3.5. Nerve Injury Repair

**Figure 7:**
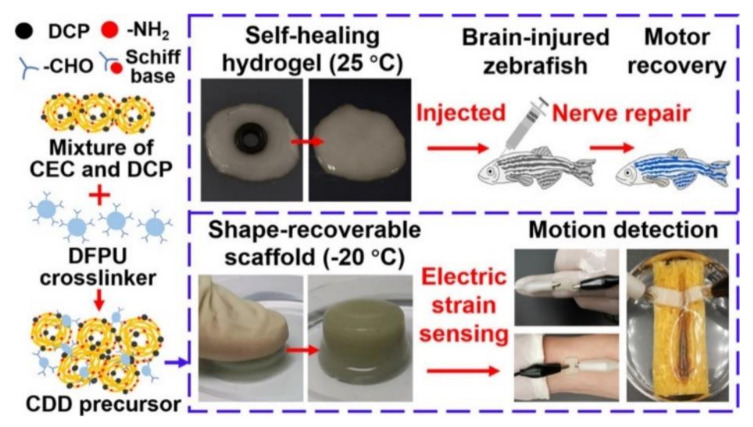
An Injectable, Electroconductive Hydrogel/Scaffold for Neural Repair and Motion Sensing [99]. Reproduced with permission from Xu, J, Chem. Mater.; published by American Chemical Society, 2020.

Nerve damage can have devastating consequences for patients, including lifelong disability and death [92,93,94]. The regenerative medicine treatment strategy is to achieve nerve repair by supplementing new nerve cells and inhibiting inflammatory response [95]. However, direct transplanted neural stem cells do not have physical support in the injured area to fill the lesion and the lack of microenvironment for cell growth and differentiation, making the survival rate of neural stem cells after transplantation extremely low. Hydrogels have adjustable physical and chemical properties to fill irregular pathological cavities in the brain and provide a favorable microenvironment for the growth and proliferation of nerve cells [96]. Self-healing hydrogels can achieve self-healing of nerves’ mechanical damage, maintain the integrated structure of nerve regeneration, and maintain long-term transplant structural integrity and persistence (Table 5) (Figure 7).

Lou J et al. [97] developed injectable self-healing hybrid hydrogels using Fmoc grafted chitosan (FC) and Fmoc peptide (FI). At the same time, curcumin embedded and physically adsorbed can also be slowly released in the FC/FI system, making FC/FI-cur hydrogel accelerate neurite outgrowth of dorsal root ganglia (DRG) neurons. WenShi et al. [98] prepared HA-PBA hydrogels by boronic ester dynamic covalent bond. The HA-PBA hydrogels can be used for reactive oxygen species responsive drug delivery and protected cells from ROS induced damage. In additionally, HA-PBA hydrogels could use for “direct-in-gel” printing to print 3D cell scaffolds.

In nerve repair, biomaterials with conductive activity show unique advantages in enhancing nerve activity and neuronal differentiation, which can significantly improve the possibility of nerve regeneration and myelin regeneration, to achieve complete overall recovery [100,101]. Biocompatible ECH dressing consisting of TA and polypyrrole(PPy) can attach to the high conductive film on the injured nerve and automatically enwrap it in the size-matched tubular structure. It forms a stable and intimate bridge coupling with the electrogenic nerve tissue to promote axonal regeneration and myelin regeneration [102].

**Table 5:** Self-healing hydrogel for nerve injury repair.

Hydrogel Substrate	Self-Healing Mechanism	Self-Healing Cycle	Inductor	Binding Mode	Mechanical Property	Characteristics of Hydrogels	Ref.
Oxidized konjac glucomannan@Amino-PEI	Schiff base	2 h	CNTs	Physical doping	more than 1 kPa (storage modulus)	pH sensitivity/ Bio-printability/ Conductivity	[103]
FC@FI	Hydrogen bond	/	Curcumin	Embedding and physical adsorption	1 kPa (storage modulus)	Injectable and self-healing properties.	[97]
*N*-carboxyethyl chitosan@aldehyde- difunctional polyurethane	Schiff base	30 min	chitosan-modified polypyrrole nanoparticle	Ion interaction	250 Pa (storage modulus)	Fast self-healing/ Tructural stability/ Durable elasticity.	[99]
L-glutamine amide derivative and benzaldehyde	Schiff base	40 s	L-DOPA	Dissolve	85 Pa (storage modulus)	Rheological property/ Self-healing.	[104]
difunctional-PEG @glycol CS	Schiff base	9 h	Cellulose nanofiber	UV crosslinking	2 kPa (storage modulus)	Biodegradable/ Tunable self-healing properties.	[105]
TA	Ion interaction	/	Pyrroles/ ferric chloride hexahydrate	Coordination bonds	846 ± 12 Pa (storage modulus)	Porous/ Conductive/ Bioadhesion	[102]
HA-PBA@PVA	Borate ester bond	10min	Neural progenitor cells	Encapsulated	1155 Pa (storage modulus)	pH sensitivity/ ROS responsive/ Anti-oxidative	[98]

## 4. Conclusions and Recommendations

Based on the self-healing mechanism of self-healing hydrogels, the design strategies of self-healing hydrogels based on dynamic covalent and noncovalent interactions are summarized and sorted. The tissue engineering applications of self-healing hydrogels in bone, cartilage, skin, cardiovascular system, and nerve were also reviewed.

The formation mechanism of self-healing hydrogels determines the basic properties of hydrogels. Such as pH sensitivity of borate ester bond hydrogels, rapid healing of hydrogen bond hydrogels and strong mechanical properties of ionic bond hydrogels, but the physiological environment is a complex system. The single self-healing hydrogel based on dynamic covalent bond or dynamic noncovalent bond interaction mechanism has the problems of weak mechanical properties, harsh self-healing conditions and incomplete recovery. In previous comments, some researchers mentioned that through various dynamic covalent or noncovalent bonds, some researchers combined solid and rigid networks with weaker networks, usually made through reversible crosslinking, to improve mechanical properties. This is a hydrogel design idea with great potential.

When designing hydrogels for application in different tissues, researchers often choose to add different factors to the hydrogel network to improve the repair effect of tissues. For example, hydroxyapatite was added in bone repair hydrogel, neural cells were added in nerve repair hydrogel, and growth factors were added in skin repair hydrogel. Specific factors can significantly improve the functionality and repair effect of hydrogels. In complex in vivo mechanical environment, the release of physical doping or encapsulation factors is not controllable, which may bring some hidden dangers. If these hydrogel factors are combined to the three-dimensional network of hydrogel through chemical bonds (not only mentioned above), perhaps these factors will provide further improvement for the performance of hydrogel.

We do not recommend that researchers simply mix or encapsulate simple physical factors in the inherent three-dimensional structural network of hydrogels in the design process. In tissue engineering applications, the physical properties of hydrogel are not the only indicator. The mechanical properties required in different tissues (such as the difference between bone and skin) are different, but the hydrogels needed to achieve various functions in the same tissues (such as bone adhesion and filling of the bone defect) are different. In addition to the natural three-dimensional structure, hydrogel can achieve a variety of tissue repair factors (stem cells, drugs, bioactive substances, etc.) with local and precise release. Researchers can also modify the components of hydrogels in the matrix design of hydrogels to make hydrogels have more intelligent responses to the external environment (pH response, light response, thermal response, etc.). Hydrogels with broad diversity and adjustability exhibit more potential than other biomaterials in tissue engineering applications. In designing hydrogels, we should prepare different functional hydrogels for different application scenarios to further open the application of hydrogels.

## Figures and Tables

**Table 4:** Self-healing hydrogel for Cardiac repair.

Intermolecular Electrostatic Interactions	Self-Healing Mechanism	Self-Healing Cycle	Inductor	Binding Mode	Mechanical Property	Characteristics of Hydrogels	Ref.
SaB-PDA/EMH	Hydrogen bonding/ Ion interaction	Not mentioned	SaB	Hydrogen bonds	28 kPa (storage modulus)	Drug slow-release/ Continuously release SaB	[87]
OA/Gelatin	Schiff base/ Hydrogen bond/ Ion interaction	Few minutes	PAA	Schiff base	1.94 kPa (storage modulus)	Mechanically tunable/ Conductive	[88]
Poly(N-isopropyl acrylamide) -4PBA@poly(vinyl alcohol)	Borate ester bond	5 min	oxidized- cellulose nanofiber	Physical mixing	0.07 Pa (storage modulus)	3D printability/ Thermoresponsive	[89]
F127-PEI@Aldehyde pullulan	Schiff base	15 s	Adipose mesenchymal stem cell	Ion interaction	About 1 kPa	Thermosensitive/Adhesive/ Antibacterial, hemostatic/ UV-shielding	[90]
Methacrylated hyaluronic acid @3-minophenylboronic acid modified sodium alginate	Borate ester bond	10 min	Bioglass (BG)	Physically doping	337 ± 45 Pa (storage modulus)	Cell delivery/ Wound healing	[91]

## Data Availability

Not applicable.
